# *Tabernaemontana stapfiana* Britten (Apocynaceae) Stem Bark Prevents Alcl_3_-Induced Cognitive Disability: Antioxidant and Anti-Inflammatory Activities in Wistar Rats

**DOI:** 10.1155/omcl/5106228

**Published:** 2025-08-07

**Authors:** Mumbi Laurantine Ngenteh, Kada Antoine Sanda, Tangu Patience Neng, Bih Belta Lilian Fubi, Ndifor Rose Nchang, Kiafon Betrand Nsah, Oumar Mahamat

**Affiliations:** Department of Zoology, Faculty of Science, The University of Bamenda, Bamenda, Cameroon

**Keywords:** cognitive impairment, inflammation, oxidative stress, *Tabernaemontana stapfiana*

## Abstract

The present study aimed to evaluate the protective effects of aqueous and ethanol extracts of *Tabernaemontana stapfiana* (*T. stapfiana*) on cognitive disability induced by aluminum chloride (AlCl_3_) in Wistar rats. Forty-five Wistar rats were distributed in different groups of five animals each. Test groups were daily given AlCl_3_ (100 mg/kg) following by the doses of the extracts for 21 days. At the end of treatment period, rats were sacrificed and the brain homogenate and serum were prepared and used to evaluate oxidative stress in brain and serum cytokines using colorimetric tests and ELISA, respectively. The findings of this study showed that reduced brain, body weight, and antioxidant enzymes (reduced glutathione [GSH], catalase [CAT], and superoxide dismutase [SOD]), while it increases oxidant biomarkers (malondiadehyde (MDA), nitric oxide (NO) and inflammatory cytokines (IL-10, TNF-α, IL-1β, and IL-6). Therefore, the administration of aqueous or ethanol extracts of *T. stapfiana* stem bark significantly (*p*  < 0.001) reduced the IL-10, TNF-α, IL-1β, and IL-6 levels in AlCl_3_-treated rats compared to non treated rats. Moreover, the extracts significantly (*p*  < 0.001) changed the oxidant–antioxidant balance by reducing the MDA and NO levels, while increasing SOD and GSH concentrations caused and NO in AlCl_3_-treated rats as compared to nontreated rats. Conclusively, aqueous or ethanol extracts of *T. stapfiana* stem bark prevented the oxidative stress and inflammation in brain, which made the brain to be not change after administration of the causative agent of cognitive impairment (CI), AlCl_3_.

## 1. Introduction

Cognitive functions refer to mental processes involved in acquiring knowledge, manipulating information, and reasoning. Cognitive functions include the domains of perception, memory, learning, attention, decision-making, and language abilities of life [1]. Preserved cognitive functioning is integral to maintaining a healthy, active, and independent lifestyle. Cognitive impairment (CI) refers to a continuum of severity from “mild” (MCI) which is a cognitive deficit that do not significantly interfere with the autonomy or social behavior of the patient and appears in psychometric assessment, to “severe” such as dementia that interferes with activities of daily life [2]. CI which is a neurodegenerative disorder affects mostly the elderly individuals with a profound decline in cognitive function and cumulative neurophysiatric changes which include alterations in mood and behavior in conjunction with memory impairments [2].

The exact mechanism(s) of neurodegeneration has not been fully described yet, however, it has been stated that the neuronal loss, especially of the cholinergic neurons and of the neuronal synapses described in neurodegeneration is related to the deposition of abnormal proteins in the cerebral cortex and other brain areas [3]. These abnormal proteins include insoluble amyloid beta (Aβ) proteins that are deposited outside the cerebral neurons forming extracellular “senile plaques” and the microtubules-associated protein (tau protein) which becomes highly phosphorylated and hence aggregated inside the neurons forming intracellular “neurofibrillary tangles” (NFTs) [2, 3]. Neurodegenerative disease is characterized pathologically by oxidative stress, neuro-inflammation and synaptic dysfunction, which may be caused in part by abnormal aggregation of senile plaques and NFTs, causing deleterious synaptic and neuronal loss which often begins several years prior to memory loss [4]. Heavy metals (HMs) are naturally occurring elements with high atomic weight their wide distribution in the environment and daily use in processing industries, house hood, technological, and medical involvement has led to a raising concern of their potential effects on human system [5]. Aluminum is one of the most widely distributed metal on the planet earth and it is used in the production of many every-day products such as food wrapping foils and cooking utensils can be a potential source of damage to humans [3]. Aluminum has an estimated half-life of elimination in the brain at > 100 days and can last for about 7 years in the human body, which is a reason for the neurodegeneration caused by aluminum [5]. Aluminum is associated with diverse nervous disorders and sstudies have determined that aluminum can lead to neuronal death by the production of ROS and by affecting mitochondria [3, 5]. Due to the long persistence of aluminum in the body it causes toxicity by generating ROS that acts against the antioxidant defense system [4]. Aluminum accumulates in organs such as the liver, brain, and kidneys where it competes with calcium for absorption, thereby, affecting skeletal mineralization [5]. Various therapeutic strategies that can slow or stop the neurodegenerative processes of toxicity are therefore based on their expected antioxidant and anti-inflammatory activities [6].

CIs like Alzheimer's disease (AD) has been managed with drugs such as Donepezil [7] and Memantine [8, 9], levodopa and carbidopa for Parkinson's diseases [10], and metformin for diabetes [11]. Due to the constraints of assisted neurodegenerative techniques, patients tend to resort to alternative remedies for CI such as neurodegenerative disease, including herbal treatment [12]. *Tabernaemontana stapfiana* is an herbaceous plant from the Apocynaceae family which is extensively used in the traditional system of medicine in many countries. Its decoction is commonly used in Cameroon as a form of treatment for CI in patients suffering of ADs. The plant extract of *T. stapfiana* was assessed in our previous study and demonstrated significant action preventing to CI like-behavior. This study is designed to evaluate the potential antioxidant and anti-inflammatory effects of aqueous and ethanol extracts of *T. stapfiana* in rats with ACl_3_-induced CI.

## 2. Materials and Methods

### 2.1. Identification and Collection Plant Material

A sample of *Tabernaemontana stapfiana* was collected in Lebialem village, South West region of Cameroon in November 2023. A specimen was taken to the national herbarium. It was identified in comparison with the collection of Thomas D.W (Number 2621) of the national herbarium (Number: 50330 SRF/Cam). After identification, the stem bark was collected, washed, and air dried for 7 days.

### 2.2. Extraction of Plant Material

The ethanolic extract was prepared by macerating 300 g of powdered stem bark of *T. stapfiana* in 1 L ethanol for 72 h at room temperature. While to have the the aqueous extract, 500 g of stem bark powder was prepared by decoction in distilled water (5 L) for 30 min and allowed to cool. The preparations were then filtered using Whatman No. 1 filter paper and the filtered was evaporated in an oven at 40°C. The dried matter obtained or extract were weigthed and kept at 4°C. Yields of 7.74% and 11.81%.for the ethanolic extract and the aqueous extract, respectively.

### 2.3. Experimental Animals

Wistar rats were obtained from the animal house of the University of Yaoundé 1, Cameroon. The animals were caged in plastic basins of dimension (48.5 cm × 33.5 cm × 22.5 cm). They were raised in the animal house at room temperature with a 12 h' light–dark cycle. They were feed with an appropriate diet (corn flour, powdered dry fish and kennel, egg shell, and multivitamins) and tap water ad libitum. They were handled according to the International Guideline for the Care and Use of Laboratory Animal published by the United States National Institutes of Health and the National Ethical Committee Guideline. The protocol was approved by Scientific Committee of Department of Zoology. All animals were acclimatized for 2 weeks before starting the experiment.

### 2.4. Animals Grouping and Treatment

After acclimatization period of 2 weeks, 45 Wistar rats were distributed in control and experimental groups of five animals each into nine different groups. Group 1: Normal (distilled water 5 mL/Kg p.o). Group 2: Negative control (AlCl_3_ only 100 mg/kg p.o). Group 3: Positive control (AlCl_3_ 100 mg/kg + Donepezil, 3 mg/kg p.o). Group 4, 5, and 6 were administered AlCl_3_ 100 mg/kg and the aqueous extract of *T. stapfiana* at 125, 250, and 500 mg/kg (p.o), respectively, whereas Group 7, 8, and 9 were administered AlCl_3_ 100 mg/kg and the ethanol extract of *T. stapfiana* at 125, 250, and 500 mg/kg (p.o), respectively. Animals of each group received the various doses of treatments orally by using an esophageal canula 1 h after the injection of AlCl_3_ (100 mg/kg). Al was administered to all the groups except the normal control (NC) group. The dose of AlCl_3_ was chosen according to literature [3], while that of Donepezil was done according to literature [7]. Treatment was administered for 21 days [13].

### 2.5. Preparation of Brain Homogenate and Serum

Animals were weighed and anesthetized by injection of the mixture of diazepam (10 mg/kg) and ketamine (50 mg/kg) [13]. Blood was collected by cardiac puncture in dried tubes, centrifuged (1000 rpm/min) and serum was collected. Skull was also dissected and the brain collected and weighed. A portion of the brain (half) was homogenized in phosphate buffer (pH 7.4, 50 mM). Serum and homogenate were stored at −4°C.

### 2.6. Biochemical Analysis

Biochemical analysis was consisted to the evaluation of the oxidants and antioxidants biomarkers and nitric oxide (NO) in brain homogenate. The evaluated parameters included superoxide dismutase (SOD), catalase activity (CAT), reduced glutathione (GSH), thiobarbituric acid reactive substance (TBARS) which were evaluated following the methods of Misra and Fridovich [14], Shina [15], Ellman [16], Wlbur et al. [17], and Gornall et al. [18]. The NO was assessed using the Greiss reagent [19].

### 2.7. Serum Cytokines Analysis

Serum levels of tumor necrosis factor alpha (TNF-α), interleukin 1-beta (IL-1β), interleukin 6 (IL-6) and interleukin 10 **(**IL-10) were determined using ELISA techniques. Specific ELISA Kits (Quantikine Colorimetric ELISA Kits (Quantikine), R&D Systems Biotech, USA) were used for the purpose. The assay was carried out according to the manufacturer's instructions.

### 2.8. Statistical Analysis

Data were presented as mean ± standard error of mean (SEM) values (*n* = 5). They were analyzed by one-way analysis of variance (ANOVA), and differences between groups assessed using Turkey. The unpaired *t*-test was further performed to distinguish between the different doses. Differences were considered statistically significant at *p*  < 0.05. All analyses were performed using Graph pad prism version 8.01.

## 3. Results

### 3.1. Effects of the Extracts of *T. stapfiana* on ACl_3_-Induced Body and Brain Weight

**Figure 1:**
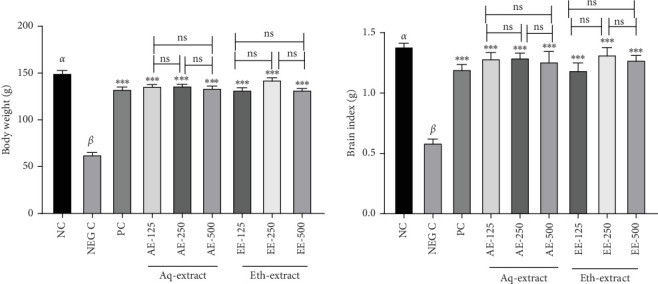
Body and brain weights post a cotreatment with ACl_3_ and the aqueous and ethanol extracts of *T. stapfiana* stem bark in rats. Barchart represent means ± standard error of means (SEM), *n* = 5 rats per group. Asterisks indicate the groups treated with extracts vs. negative control. *α* and *β* indicate negative control vs. positive control difference. ns: indicate not significant at *p*  > 0.05. NC = normal control, NEGC = negative control, PC = positive control.

Figure 1 presents the effect of the aqueous and ethanol extracts of *T. stapfiana* on aluminum-induced body and brain weight of the animals. Negative control (NEG C) group showed significant reduction in body and brain weight compared to the NC group. But, the body and brain weights of rats given ACl_3_ following by the extracts of *T. stapfiana* did not decrease significantly compared to negative control.

### 3.2. Effects of the Aqueous and Ethanol Extracts of *T. stapfiana* Stem Bark on ACl3-Induced Brain Oxidative Stress

As shown in Table 1, the NEG C group that were orally given AlCl_3_ showed significant increase of CAT activity and levels of MDA and NO, while there was a significant decrease in SOD activity, GSH compared to the NC group. In one site, the administration of the aqueous and ethanolic extracts of *T. stapfiana* (125, 250, and 500 mg/kg) significantly decreased the levels of CAT activity, NO, and MDA, in the other hand increased the SOD and GSH levels in the animals given AlCl_3_.

### 3.3. Effects of the Aqueous and Ethanol Extracts of *T. stapfiana* Stem Bark on Inflammatory Cytokines in ACl_3_-Treated Rats

Table 2 summarizes the effect of the aqueous and ethanol extracts of *T. stapfiana* against AlCl_3_ effect on some inflammatory cytokines. AlCl_3_ (NEG) administration significantly (*p*  < 0.001) reduced IL-10 level while caused an increase in TNF-α, IL-1β, and IL-6 levels compared to the NC group. The administration of extract (aqueous and ethanolic) at 125, 250, and 500 mg/kg significantly corrected the changes of the inflammatory cytokines caused by AlCl_3_ by increasing IL-10 and decreasing TNF-α, IL-1β, and IL-6 compared to the NEG C).

## 4. Discussions

Measurement of organ weight (brain) is important in assessment of drugs effectiveness. The administration of AlCl_3_ significantly affects weights of the brain, which the cotreatment with the extracts of *T. stapfiana* prevented. The brain plays an important role in learning, perception, and memory, and AlCl_3_ was reported to affect nervous physiology resulting in CI [20]. Therefore, this may be an evidence that the extracts of *T. stapfiana* possibly due to the presence of important natural compounds such as polysaccharides, glycoproteins, and vitamins [21], are beneficial in treatment or prevention of the nervous effect of AlCl_3_, notably CI.

Therapeutic strategies that slow or stop the neurodegenerative processes of toxicity are expected to have a major impact on the treatment of neurological problems [6, 22]. The hypothesis about the mechanisms by which neurons come into necrotic or apoptotic processes has led to belief that the therapeutic use of antioxidants plays a beneficial in aging and neurodegenerative disorders [22]. Previous studies have determined that aluminum can lead to neuronal death by the production of ROS and by affecting mitochondria [3]. Results of this study showed that AlCl_3_ significantly decreased the brain SOD, the level of GSH, but increases CAT activity, NO, and MDA levels, demonstrating that AlCl_3_ increases the level of reactive oxygen species (ROS) in brain.

In brain, GSH plays an important role to prevent the damage of neurons [8]. The brain is particularly susceptible to oxidative insults and its antioxidant defense system is dependent on its GSH content. GSH level was significantly decreased in AlCl_3_-induced rats, while it was alleviated by the aqueous and ethanol extracts of *T. stapfiana* with the dose of 250 mg/kg alleviated the decrease in GSH, showing the extracts can prevent the effect the neurological increase in ROS [3].

SODs form the front line of defense against ROS-mediated injury [23] and constitute a very important antioxidant defense against oxidative stress in the body. The administration of AlCl_3_ leads to a decrease in SOD activity. This low level SOD showed caused by AlCl_3_ was significantly alleviated by administration of the aqueous and ethanol extracts of *T. stapfiana*. This finding demonstrates the effectiveness of the extracts in treatment of neurodegenerative impairment due to increase in ROS. This result coincides with that of Simplice et al [23].

TBARSs are a common way to measure lipid peroxidation products in cells, tissues, and body fluids. Previously, study reported that AlCl_3_ cause neurodegeneration or CI by increasing the TBARS in temporal lobe homogenates [24]. Also, in this study, AlCl_3_ significantly increased the level of MDA, which the co-administration of the aqueous and ethanol extracts of *T. stapfiana* prevented, indicating that extracts have preventive effect against CI.

Nitrite oxide is known to play an important role in the neurotoxic actions of glutamate and trans-synaptic regulation, as well as in learning and memory processes. Concentration of NO is generally high in person with a cognitive or other neurodegenerative [3]. as it is shown in the rats that were given AlCl_3_. Results, therefore, showed that the administration of *T. stapfiana* extracts has reduced the concentration of NO at the brain level. The decrease of NO in rat taken the AlCl_3_ following of the extracts demonstrate that the extracts are beneficial in control of CI.

In AlCl_3_-induced like CI, it was reported that AlCl_3_ increases proinflammatory cytokines, such as IL-1β, IL-6, or TNF-α and decreases IL-10 [25, 26]. Cytokines have been long known as mediators and inhibitors of neurodegeneration. TNF-α might contribute to neuronal injury and exert protective effects [27]. It has been demonstrated that the increase in TNF-α levels induced by AlCl_3_ is associated with the nuclear factor kappa-β (NF-kappa β) activation and inducible NO synthase (iNOS) expression in the brain cortex that accounts for the neurodegenerative changes [28–30]. Besides, study demonstrated AlCl_3_ stimulates the secretion of pro-inflammatory cytokines [8]. Results of study showed that the administration of *T. stapfiana* extracts has reduced the increase in the IL-1β, IL-6, and TNF-α levels caused by AlCl_3_ in rats demonstrating the importance of the *T. stapfiana* extracts in control of neuroinflammation and eventually CI. While the current study provides strong biochemical evidence of neuroprotection through attenuation of oxidative stress and neuroinflammation, we acknowledge the absence of direct behavioral assessments of cognitive function, such as spatial learning or memory tasks. These tests are vital in fully characterizing cognitive outcomes in preclinical models of neurotoxicity. Therefore, future studies will incorporate behavioral testing to corroborate the cognitive protective effects of *T. stapfiana* observed at the molecular level in this study.

## 5. Conclusions

In summary, the findings of this study demonstrate that the aqueous and ethanol extracts of *T. stapfiana* possessed an antioxidant activity by regulating the SOD, MDA, GSH, CAT, and NO levels. These extracts also showed anti-inflammatory activities through the reduction of the increase in the IL-1β, IL-6, and TNF-α levels. Both the antioxidant and anti-inflammatory activities of the aqueous and ethanol extracts of *T. stapfiana* justify the effectiveness in control of CI as well as neurodegenerative diseases in general.

## Figures and Tables

**Table 1:** Reduced glutathione (GSH), catalase (CAT), superoxide dismutase (SOD), malondiadehyde (MDA) and nitric oxide (NO) levels post a cotreatement with ACl_3_ and the aqueous and ethanol extracts of *T. stapfiana* stem bark in rats.

Parameters	Extract	Controls			Extracts doses			F; *p*-Value
		NC	NEG	PC	125 mg/kg	250 mg/kg	500 mg/kg	
SOD activity (% I)	Aqueous	33.65 ± 2.83^≠^	5.03 ± 0.63	19.81 ± 2.83^€^	25.79 ± 1.31^€^	22.64 ± 0.36^€^	33.96 ± 2.02^≠^	F_(5, 9)_ = 32.95; *p* < 0.001
	Ethanol	33.65 ± 2.83^≠^	5.03 ± 0.63	19.81 ± 2.83^€^	23.69 ± 1.64^€^	26.00 ± 1.37^€^	31.87 ± 0.91^≠^	F_(5, 9)_ = 31.40; *p* < 0.001

CAT (µmole/H_2_O_2_)	Aqueous	1.08 ± 0.03^≠^	22.08 ± 0.23^ns^	2.17 ± 0.13^≠^	1.02 ± 0.05^≠^	1.08 ± 0.01^≠^	1.5 ± 0.06^≠^	F_(5, 10)_ = 5974; *p* < 0.001
	Ethanol	1.08 ± 0.03^≠^	22.08 ± 0.23^ns^	2.17 ± 0.13^≠^	0.81 ± 0.04^≠^	0.92 ± 0.02^≠^	3.27 ± 0.1^≠^	F_(5, 9)_ = 5356; *p* < 0.001

GSH (µmole/mL)	Aqueous	1.62 ± 0.02^≠^	0.17 ± 0.03	0.89 ± 0.02^≠^	0.88 ± 0.02^≠^	1.11 ± 0.05^≠^	0.78 ± 0.01^≠^	F_(5, 9)_ = 216.5; *p* < 0.001
	Ethanol	1.62 ± 0.02^≠^	0.17 ± 0.03	0.89 ± 0.02^≠^	1.2 ± 0.01^≠^	1.1 ± 0.01^≠^	1.3 ± 0.04^≠^	F_(5, 9)_ = 33.45; *p* < 0.001

MDA (µmole/mL)	Aqueous	3.09 ± 0.10^≠^	21.14 ± 0.18	2.80 ± 0.42^≠^	2.92 ± 0.74^≠^	1.69 ± 0.03^≠^	2.42 ± 0.05^≠^	F_(5, 9)_ = 2215; *p* < 0.001
	Ethanol	3.09 ± 0.10^≠^	21.14 ± 0.18	2.80 ± 0.42^≠^	2.03 ± 0.08^≠^	2.85 ± 0.27^≠^	2.80 ± 0.03^≠^	F_(5, 9)_ = 1147; *p* < 0.001

Nitrite (µmole/mL)	Aqueous	4.48 ± 0.09^≠^	0.91 ± 0	3.79 ± 0.03^≠^	3.63 ± 0.02^≠^	4.01 ± 0.09^≠^	4.1 ± 0.07^≠^	F_(5, 9)_ = 326.1; *p* < 0.001
	Ethanol	4.48 ± 0.09^≠^	0.91 ± 0	3.79 ± 0.03^≠^	3.59 ± 0.03^≠^	3.92 ± 0^≠^	3.83 ± 0.05^≠^	F_(5, 9)_ = 693.7; *p* < 0.001

*Note:* Values represent means ± standard error of means (SEM), *n* = 5 rats per group. (^¥^) differ significantly at *p* < 0.05; (^€^) differ significantly at *p*  < 0.01; (^≠^) differ significant at *p*  < 0.001 and (ns) indicate not significant at *p*  > 0.05 compared to the negative control.

Abbreviations: NC, normal control; NEGC, negative control; PC, positive control.

**Table 2:** IL-10, TNF-α, IL-1β, and IL-6 levels post a cotreatement with ACl_3_ and the aqueous and ethanol extracts of *T. stapfiana* stem bark in rats.

Parameters	Extract	Controls			Extracts doses			F; *p*-Value
		NC	NEG C	PC	125 mg/kg	250 mg/kg	500 mg/kg	
TNF-α (pg/mL)	Aqueous	150.6 ± 6.45^≠^	364.07 ± 7.57	156.61 ± 2.77^≠^	342.4 ± 1.72^€^	310.2 ± 3.14^≠^	273.3 ± 7.31^≠^	F_(5, 21)_ = 568; *p* < 0.001
	Ethanol	150.6 ± 6.45^≠^	364.07 ± 7.57	156.61 ± 2.77^≠^	369.39 ± 15.48^€^	279.5 ± 11.33^≠^	295.494.16^≠^	F_(5, 21)_ = 273.9; *p* < 0.001

IL-1β (pg/mL)	Aqueous	184.77 ± 6.53^≠^	360.09 ± 4.54^ns^	159.66 ± 6.75^≠^	327.4 ± 6.38^≠^	265.3 ± 9.98^≠^	178.26 ± 6.19^≠^	F_(5, 21)_ = 314.0; *p* < 0.001
	Ethanol	184.77 ± 6.53^≠^	360.09 ± 4.54^ns^	159.66 ± 6.75^≠^	333.06 ± 13.14^≠^	279.5 ± 5.73^≠^	173.68 ± 8.39^≠^	F_(5, 21)_ = 275.2; *p* < 0.001

IL-6 (pg/mL)	Aqueous	329.37 ± 9.42^≠^	487.61 ± 10.07	356.97 ± 6.06^≠^	450.59 ± 13.65^¥^	391.92 ± 17.71^≠^	349.4 ± 6.18^≠^	F_(5, 21)_ = 68.58; *p* < 0.001
	Ethanol	329.37 ± 9.42^≠^	487.61 ± 10.07	356.97 ± 6.06^≠^	460.12 ± 15.72^¥^	408.96 ± 7.02^≠^	373.52 ± 4.51^≠^	F_(5, 21)_ = 117.1; *p* < 0.001

IL-10 (pg/mL)	Aqueous	168.54 ± 12.38^≠^	392.55 ± 5.38^ns^	164.02 ± 4.08^≠^	371.99 ± 3.83^¥^	284.22 ± 9.99^≠^	237.55 ± 6.76^≠^	F_(5, 21)_ = 446.6; *p* < 0.001
	Ethanol	168.54 ± 12.38^≠^	392.55 ± 5.38^ns^	164.02 ± 4.08^≠^	370.05 ± 12.11^¥^	274.85 ± 14.25^≠^	252.34 ± 11.72^≠^	F_(5, 21)_ = 207.4; *p* < 0.001

*Note:* Values represent means ± standard error of means (SEM), *n* = 5 rats per group. (^¥^) differ significantly at *p* < 0.05; (^€^) differ significantly at *p*  < 0.01; (^≠^) differ significant at *p*  < 0.001, and (ns) indicate not significant at *p*  > 0.05 compared to the negative control.

Abbreviations: NC, normal control; NEGC, negative control; PC, positive control.

## Data Availability

All the data for this manuscript are available by requesting the corresponding author.
